# Practical Calling Approach for Exome Array-Based Genome-Wide Association Studies in Korean Population

**DOI:** 10.1155/2015/421715

**Published:** 2015-12-27

**Authors:** Tae-Joon Park, Lyong Heo, Sanghoon Moon, Young Jin Kim, Ji Hee Oh, Sohee Han, Bong-Jo Kim

**Affiliations:** Division of Structural and Functional Genomics, Center for Genome Science, National Institute of Health, Centers for Disease Control and Prevention, Chungcheongbuk-do 363-700, Republic of Korea

## Abstract

Exome-based genotyping arrays are cost-effective and have recently been used as alternative platforms to whole-exome sequencing. However, the automated clustering algorithm in an exome array has a genotype calling problem in accuracy for identifying rare and low-frequency variants. To address these shortcomings, we present a practical approach for accurate genotype calling using the Illumina Infinium HumanExome BeadChip. We present comparison results and a statistical summary of our genotype data sets. Our data set comprises 14,647 Korean samples. To solve the limitation of automated clustering, we performed manual genotype clustering for the targeted identification of 46,076 variants that were identified using GenomeStudio software. To evaluate the effects of applying custom cluster files, we tested cluster files using 804 independent Korean samples and the same platform. Our study firstly suggests practical guidelines for exome chip quality control in Asian populations and provides valuable insight into an association study using exome chip.

## 1. Introduction

A genotyping array is a cost-effective and efficient platform for the identification of variants in a large population [1, 2]. Until now, genome-wide association studies (GWASs) have identified large numbers of variants associated with complex diseases using genotyping arrays [3]. Most identified variants are among the common variants (minor allele frequency (MAF) ≥ 0.05) found throughout the population, even though detection of rare genetic variants in human populations is an important aspect of understanding pathophysiological variability in complex diseases [4, 5]. Next-generation sequencing technologies are effective methods to detect rare variants in the human genome [2], but these technologies have higher cost per sample compared with a genotyping array. Also, because next-generation sequencing has a high noise-to-signal ratio, experimental validation is necessary [6]. Exome-based genotyping arrays, such as the Illumina Infinium HumanExome BeadChip (hereafter referred to as exome chip) and the Affymetrix Axiom exome array, were recently introduced. Prices of these platforms are lower than for other kinds of genotyping arrays. Moreover, exome-based genotyping has the capacity to discover rare variants in exon regions associated with complex diseases [7–9].

Genotype calling algorithms based on clustering methods are vulnerable to detecting rare variants. At times, clustering of rare or low-frequency variants results in spurious genotype calls. The number of variants remaining after quality control process can be different according to diverse genotype calling methods. In addition, because of racial differences in allele frequencies of polymorphic variants [10], criteria for variants selection should be different according to ethnicities of populations.

Grove et al. proposed a list of best practices for genotype calling for exome chip methods following a study with 62,266 participants from seven genotyping centers included in the Cohorts for Heart and Aging Research in Genomic Epidemiology (CHARGE) consortium [11]. This genotype calling method suggested based on CHARGE consortium presented criteria for identified variants that may contain clustering errors. Also, this study was largely based on western populations, as those of African American (*n* = 13,605), Caucasian (*n* = 43,869), and Hispanic ancestry (*n* = 2,129) were represented in relatively large numbers, whereas only 777 Asian samples of 62,266 were analyzed. The subjects recruited for exome chip design listed in a web site, Exome Chip Design-Genome Analysis Wiki (http://genome.sph.umich.edu/wiki/Exome_Chip_Design), were also mainly comprised of European ancestry, but a small fraction of Asians including 327 Chinese individuals. In addition, Guo et al. have suggested a protocol for exome chip data consisting of 39,000 samples mainly composed of European and African ancestry [12]. Therefore, additional information from Asian samples is needed to provide efficient background data for genotype calling for Asian populations.

Here, we propose a practical method for accurate genotype calling using exome chip technologies with data from 14,647 Korean samples. Further, we provide workflow for manual reclustering and present a change in genotype calling quality following the use of custom cluster files.

## 2. Materials and Methods

### 2.1. Data Description

We performed a two-stage analysis as part of a novel genotype calling method. In the first stage, we established an efficient genotype calling process for 14,647 Korean-based data points and built a custom cluster file using quality control process that included manual reclustering for low-frequency cluster data. The Korean individuals belong to three cohorts that comprise the Korean Genome Epidemiology Study (KoGES): Ansan/Ansung (*n* = 8,012), Health Examinee (HEXA, *n* = 3,448), and the Cardiovascular Association (CAVAS) cohort (*n* = 3,187). In the second stage, we applied knowledge from our custom cluster file to independent samples, namely, 804 Korean diabetic retinopathy samples recruited from the Seoul National University Bundang Hospital.

### 2.2. Ethics Statement

Written informed consents were provided to all of the participants in this study. The study was approved by the Institutional Review Board of Korea Centers for Disease Control and Prevention and Seoul University Bundang Hospital.

### 2.3. Overall Workflow and Manual Clustering

All 14,647 Korean subjects were genotyped with an exome chip. Each genotype was automatically clustered once by Illumina GenomeStudio v2011.1 software, and then we applied criteria that have been used in the CHARGE consortium for manual reclustering and visual inspection. Parameters adjusted for criteria when using GenomeStudio software are listed in Table 1.

When we applied criteria provided by the CHARGE consortium, over half of variants were identified to be required for manual reclustering. In response, we adjusted a few criteria scores to fit our data set. Call frequency rates in all categories decreased from 1 to 0.99 and MAF in the AB category cluster error increased from 0 to 0.0002 (Table 1). Subsequently, variants that needed manual reclustering were selected by the adjusted criteria.

The customized cluster files (^*∗*^.egt), which have cluster information containing manual reclustering criteria, were generated by GenomeStudio. Sample and variant quality control of reclustered genotype data was performed following extraction of data using the PLINK format [13]. We rearranged three types of misclassified clusters using GenomeStudio as follows: (1) small number of variants misclassified with the AB genotype (Supplementary Figure  2a in Supplementary Material available online at http://dx.doi.org/10.1155/2015/421715), (2) some misclassified variants with a lower Norm *R* score and wide Norm Theta distribution (Supplementary Figure  2b), and (3) a short range for a cluster boundary (Supplementary Figure  2c).

To test the effects of our customized cluster file, we performed automated clustering on patient data for 804 Koreans with diabetic retinopathy using a customized cluster file instead of the default cluster file. Then, we compared results from default and customized cluster files.

### 2.4. Quality Control of SNPs and Samples

We used PLINK for quality control for both samples and genotypes. First, relatives defined based on identity-by-descent and samples with sex inconstancies were excluded through the PLINK options of “- -genome” and “- -check-sex”, respectively. Also, samples with low call rate (<99%) were thrown out by option “- -mind”. Next, PLINK option “- -geno 0.95 (call rate < 95%)” was used to remove variants with completely missing data or having a low call rate. Also, additional variants were excluded based on deviation from Hardy-Weinberg Equilibrium (HWE), a below-threshold *P* value (HWE *P* < 0.000001), and minor allele count (MAC) < 2. Duplicated variants were removed.

### 2.5. Overall Accuracy, Accuracy of Heteroallele, and Nonreference Concordance between Exome Chip and Whole-Exome Sequencing Data

For performance comparisons of manual reclustering, we compared exome chip data with or without reclustering process to whole-exome sequencing data. Exome chip data were extracted to PLINK format and quality control process was performed as described above. And whole-exome sequencing data for 185 samples which were the same as our exome chip samples was produced with the IlluminaHiseq2000 platform (70x coverage). These data were analyzed with the Genome Analysis ToolKitv2 (GATKv2) pipeline. Also off-target variants of whole-exome sequencing data were removed from each exome chip data for further analysis. In addition, overall accuracies of heteroalleles and nonreference concordances were calculated by our in-house customized python script.

### 2.6. Annotation

Exome chip variants were annotated with Annovar software (http://www.openbioinformatics.org/annovar). The annotation data files needed for running Annovar can be downloaded by option “- -downdb”. Supplementary Table  2 shows the number of variants within each functional category.

## 3. Results

### 3.1. Characteristics of Genotypes

A total of 242,766 genotypes were converted to PLINK format. MAF of all genotypes was calculated using option “- -freq” within the PLINK tool.  Table 2 summarizes the proportion of genotype by MAF interval of each cohort. Monomorphic variants accounted for >70% of those found in HEXA (*n* = 3,448) and CAVAS (*n* = 3,187) samples. From all 14,647 samples, we found that 66% of the data was accounted for by monomorphic variants. This percentage was slightly different than that found for the 777-sample CHARGE data set.

### 3.2. Quality Control

With regard to sample selection, samples were excluded through the quality control process as follows: (1) samples that were estimated as relatives based on identity-by-descent (*n* = 579), (2) sex inconsistency (Fail, *n* = 55), and (3) low call rate per sample (<99%, *n* = 61). Regarding variant selection, variants which are monomorphic with MAF > 0.001% (*n* = 162,757), deviated from Hardy-Weinberg Equilibrium (*P* value < 10^−6^, *n* = 1,185), and with low call rate per genotype (<95%, *n* = 24) were excluded. Consequently, 77,204 SNPs of 14,056 samples remained.

### 3.3. Genotype Calling Accuracy of the Exome Chip

To evaluate the variant calling accuracy of our method, we calculated overall concordance with frequency rates between exome chip and whole-exome sequencing data. After quality control process, a total of 78,114 and 77,204 variants remained before and after manual reclustering, respectively. Consequently, among those, 29,959 and 29,850 from before and after reclustering, respectively, were extracted for comparing performance. Mean and standard deviation of overall accuracy rate were calculated by MAF categories such as common, low-frequency, rare, and all variants. Overall accuracy rate showed that results of manual reclustering were more accurate than those of absence of manual reclustering (Table 3). Particularly, overall concordance of common variants was improved from 0.9730 to 0.9856 (Table 3).

Moreover, to evaluate the efficiency of identifying rare variants, we calculated nonreference concordance and accuracy of heteroalleles on extreme rare variants by MAC ranging from 2 to 5. Overall, nonreference concordance of after reclustering was similar to those of before reclustering. But with regard to accuracy of heteroalleles by MAC, accuracy of after quality control was significantly improved compared to those of before quality control (Supplementary Table  1).

### 3.4. Comparison of Custom (^*∗*^.egt) and Default Cluster File

**Figure 1:**
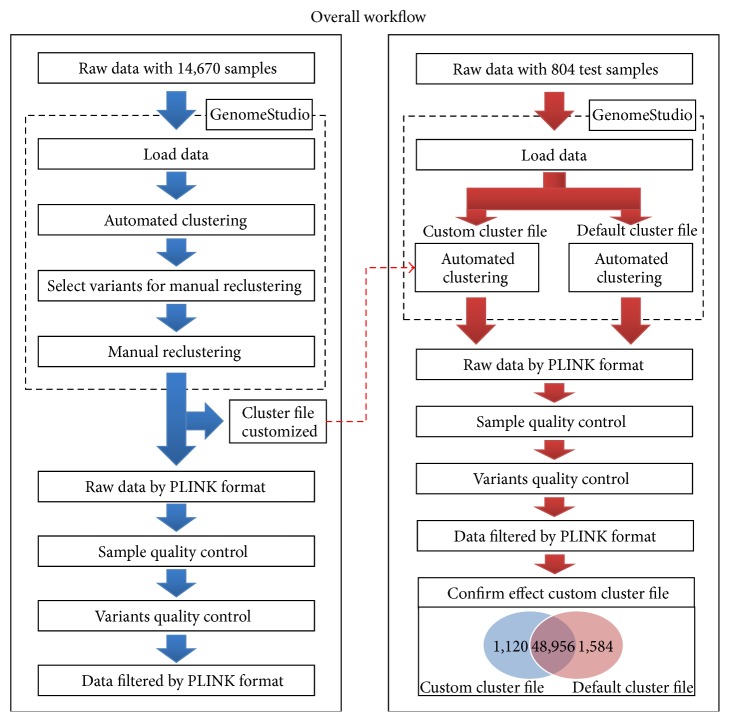
Overall workflow for genotype calling using Korean exome chip data. Left panel shows data processing and analysis for 14,647 Korean samples. Right panel shows the workflow for 804 Korean samples to test the effects of customized clustering file. The Venn diagram in right panel describes the discrepancies between custom and default cluster files tested using exome chip data from 804 Korean diabetic retinopathy samples. The number in red and blue circle of Venn diagram indicates the number of variants when adjusting default and custom cluster file, respectively.

To examine discrepancies between custom versus default cluster files, we used 804 additional Korean samples. For variants and sample selection, we used the same quality control criteria as that in the first stage of analysis. From the quality control process, the remaining number of variants using custom and default cluster files included 50,076 and 50,540 variants, respectively. A total of 48,956 variants were shared between the two cluster files, whereas 1,120 and 1,584 variants were uniquely found in the custom versus default cluster file, respectively (Figure 1). Supplementary Figure  2 shows that 1,120 misclassified variants were reclustered using our custom cluster file.

### 3.5. Functional Categories of Selected Variants

Using Annovar, we examined the discrepancy of the functional category of variants comparing data from before and after we applied our quality control guidelines. Supplementary Table  2 shows Annovar results with 15 functional categories. The rate of exonic variants using our guidelines (87.13%) was less than that seen implementing our guidelines (77.31%), whereas the rate of nongenic (intergenic and intronic) variants found using our guidelines was higher than that before implementing our guidelines (Supplementary Table  2). Moreover, relative proportions of splicing and exonic-splicing variants using our guidelines decreased by 0.39% and 0.67%, whereas the abundances of other variants increased. Supplementary Figure  1 shows the distribution of variants on each chromosome; variants found using our guidelines were not biased to a particular chromosome.

## 4. Discussion

Exome chip methodologies constitute a unique platform to perform large-scale GWASs. Not only is the price of an exome chip lower than that of other kinds of genotyping arrays, but also an exome chip focuses on the discovery of rare variants in exonic regions, which can be associated with complex diseases. The CHARGE consortium reported best approaches for genotype calling related to the HumanExome BeadChip [11]. They suggested that a custom cluster file (^*∗*^.egt) be applied to attain more accurate genotype data. However, because the HumanExome BeadChip was developed based on the genetics of a Caucasian population, substantial variants were monomorphic when we conducted GWASs using other ethnic samples, such as Koreans (Table 2). Therefore, proper genotype calling guidelines that consider ethnic differences can be important attributes of large-scale GWASs using exome chips.

Here, we propose a suitable exome chip genotype calling process to apply to Korean samples. To reduce the number of variants that were excluded by the automated genotype calling method, we paid attention to two issues in the process of quality control.

First, we adjusted several scores of automated clustering criteria such as call frequency rate and MAF of AB cluster error. When we applied automated clustering criteria recommended by the CHARGE consortium, more than half of variants were identified to be requiring manual reclustering using those filters (Table 1). Therefore, remaining data were too small to conduct a GWAS with respect to rare variants which can be a cause of rare diseases. To resolve this problem, we lowered several cut-offs. Through this process the number of excluded variants decreased by 46,070 variants (Table 1). However, the increased number of remaining variants due to lowered quality control threshold compared to CHARGE consortium guideline did not affect overall accuracy of variants (Table 3 and Supplementary Table  1).

Second, misclassified variants were rearranged by manual reclustering. We remedied three types of representative misclassification patterns, which could be reclassified among 46,070 variants (Supplementary Figure  2). As shown in Supplementary Figure  2, our correction process was robust enough to detect low-frequency and rare variants. From this correction process, 5,038 variants were restored as additional variants. Consequently, 77,204 variants were used as background data within the customized cluster file (^*∗*^.egt).

To evaluate the accuracy of the genotype call, we compared concordance rates between exome chip and whole-exome sequencing data obtained from the 185 same individuals. More than 99% of variants were the same genotype as each other (Table 3). Particularly, the concordance rate for rare variants was close to 99.9%, supporting the robustness of our data for rare variants.

We applied the customized cluster file (^*∗*^.egt) to genotype calling for 804 independent individuals. When we compared the remaining variants using the custom cluster file to those using the default cluster file, 48,956 variants were shared between the cluster files. Only 1,120 variants were uniquely found in our custom cluster files. The fact that we identified a relatively small number of variants may be attributable to the relatively small sample size with respect to finding a rare variant. Therefore, it remains possible that our customized cluster file would identify a larger number of variants if applied to larger sample sizes. In addition, we examined the number of functional variants before and after reclustering. As shown in Supplementary Table  2, we could not find bias of the number of functional variants after our approach.

Taken together, we think that our approach can provide larger number of variants with high accuracy for association analysis in Korean exome chip data. Moreover, prior knowledge data (^*∗*^.egt) can be valuable reference information for performing variant clustering of exome chip data. Our study can provide practical quality control guidelines for exome array-based GWAS using Korean population.

Although there is a limitation due to the recruitment of Korean samples only, we firstly tried to apply alternative criteria for quality control of exome chip data produced with a large scale of Asian population (more than 10,000 individuals). Consequently, we could substantially improve the call rate of Asian-specific rare variants. Considering similar allele distributions between Koreans and the Chinese/Japanese, our alternatively suggested criteria using a large scale of Korean population may be applied to other East Asian populations. Further investigations might be needed whereas our criteria for quality control would be applied to other Asian ancestry, such as Southeastern Asians.

## 5. Conclusion

We suggest practical guidelines for exome chip quality control for variant analysis in Korean populations. We also provide comprehensive evaluation of the results using whole-exome sequencing data. Moreover, ours is the first study to describe the practical use of variant calling and genotype clustering in Korean populations. Our study allows an efficient method to detect exonic variants as well as low-frequency and rare variants. Furthermore, the modified criteria for genotype quality control and clustering we suggested might be possible to be extensively applied to other East Asian populations.

## Supplementary Material

Supplementary material is composed of two supplementary tables and two supplementary figures. Supplementary tables indicate non-reference concordance and heteroallele accuracies on extremely rare variants and distributions of variants according to functional categories. Supplementary figures describe the distribution of exonic variants in each chromosome and the examples of manual re-clustering of variants.

## Figures and Tables

**Table 1:** The number of variants that were excluded after implementing automated clustering guidelines in Illumina GenomeStudio. We compared criteria of the CHARGE consortium with adjusted criteria in our study using Korean samples. The total number of SNVs was counted with redundancy. Different categories of our guideline are shown in boldface.

Type	CHARGE consortium guidelines		Our guidelines	
	Criteria	# of SNVs	Criteria	# of SNVs
Clustering errors	Call Freq 0.95~0.99	2,841	Call Freq 0.95~0.99	2,841
	Cluster Sep < 0.4	693	Cluster Sep < 0.4	693
	AB Freq > 0.6	1	AB Freq > 0.6	1
	AB *R* Mean	645	AB *R* Mean	645
	Het Excess > 0.1	13	Het Excess > 0.1	13
	Het Excess < −0.9	17	Het Excess < −0.9	17
	**MAF < 0.0001 & Call Freq ≠ 1**	**119,896 **	**MAF < 0.0001 & Call Freq < 0.99**	**2,171 **

AA cluster error	AA *T* Mean 0.2~0.3	759	AA *T* Mean 0.2~0.3	759
	AA *T* Dev > 0.025	2,195	AA *T* Dev > 0.025	2,195
	**AA Freq = 1 & Call Freq < 1**	**43,012 **	**AA Freq = 1 & Call Freq < 0.99**	**561 **

AB cluster error	AB *T* Mean 0.2~0.3,	847	AB *T* Mean 0.2~0.3,	847
	AB *T* Mean 0.7~0.8	2,685	AB *T* Mean 0.7~0.8	2,685
	AB *T* Dev ≥ 0.07	272	AB *T* Dev ≥ 0.07	272
	**AB Freq = 0 & MAF > 0**	**70,597 **	**AB Freq = 0 & MAF > 0.0002**	**11,572 **

BB cluster error	BB *T* Mean 0.7~0.8	690	BB *T* Mean 0.7~0.8	690
	BB *T* Dev > 0.025	2,742	BB *T* Dev > 0.025	2,742
	**BB Freq = 1 & Call Freq < 1**	**16,352 **	**BB Freq = 1 & Call Freq < 0.99**	**92 **

Total # of SNVs		264,257		46,076

SNV: single nucleotide variation; MAF: minor allele frequency.

**Table 2:** Genotype ratio based on minor allele frequency (MAF) interval.

MAF interval	All (*n* = 14,647)	KARE (*n* = 8,012)	HEXA (*n* = 3,448)	CAVAS (*n* = 3,187)
0	66.70%	70.43%	74.32%	74.47%
(0–0.001]	14.66%	10.98%	6.97%	6.89%
(0.001–0.005]	3.21%	3.18%	3.28%	3.19%
(0.005–0.01]	1.50%	1.50%	1.49%	1.52%
(0.01–0.05]	2.98%	2.97%	3.00%	2.98%
>0.05	10.95%	10.95%	10.94%	10.94%

**Table 3:** Overall concordance rates between before and after reclustering process based on minor allele frequency (MAF).

Categories by MAF	Overall concordance^*∗*^	
	Before	After
All	0.9881 ± 0.06	0.9928 ± 0.03
Common^1^	0.9730 ± 0.09	0.9856 ± 0.40
Less common^2^	0.9951 ± 0.04	0.9970 ± 0.02
Rare^3^	0.9983 ± 0.03	0.9994 ± 0.10

^1^MAF ≥ 5%, ^2^MAF ≥ 1% and <5%, and ^3^MAF < 1%.

^*∗*^Overall concordances were designated as mean ± standard deviation (SD).
