# Supplementary material for: Practical Calling Approach for Exome Array-Based Genome-Wide Association Studies in Korean Population
Source: Int J Genomics. 2015 Dec 27;2015:421715. doi: 10.1155/2015/421715 (PMC4706946; doi:10.1155/2015/421715)
Supplement: Supplementary file 1 — Supplementary material is composed of two supplementary tables and two supplementary figures. Supplementary tables indicate non-reference concordance and heteroallele accuracies on extremely rare variants and distributions of variants according to functional categories. Supplementary figures describe the distribution of exonic variants in each chromosome and the examples of manual re-clustering of variants. [file 421715.f1.pdf]

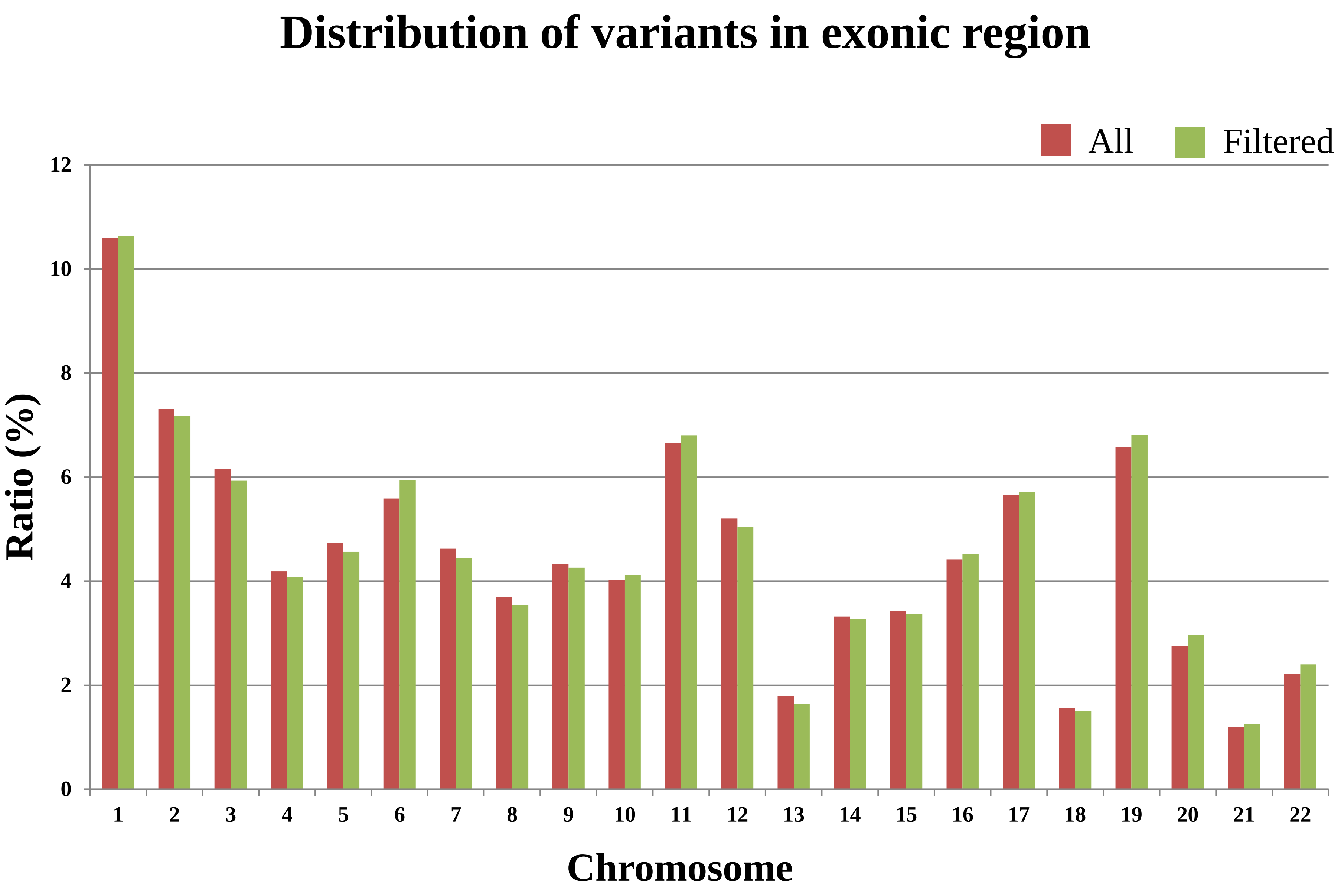
Distribution of variants in exonic region
All
Filtered
Ratio (%)
0
2
4
6
8
10
12
1
2
3
4
5
6
7
8
9
10
11
12
13
14
15
16
17
18
19
20
21
22
Chromosome

(a)

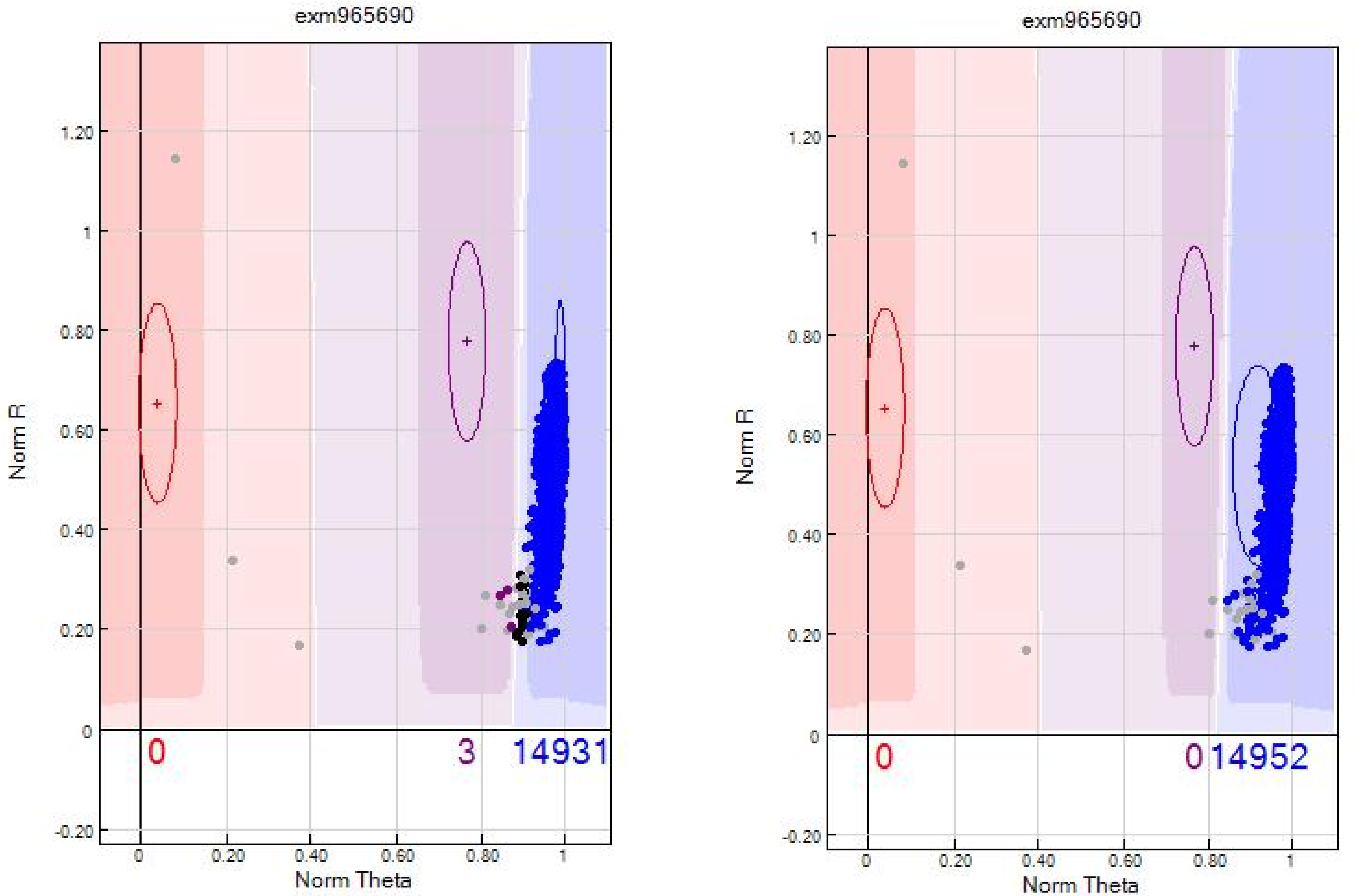
exm965690
Norm R
Norm Theta
1.20
1
0.80
0.60
0.40
0.20
0
-0.20
0.20
0.40
0.60
0.80
0
3
14931
exm965690
Norm R
Norm Theta
0
0 14952

(b)

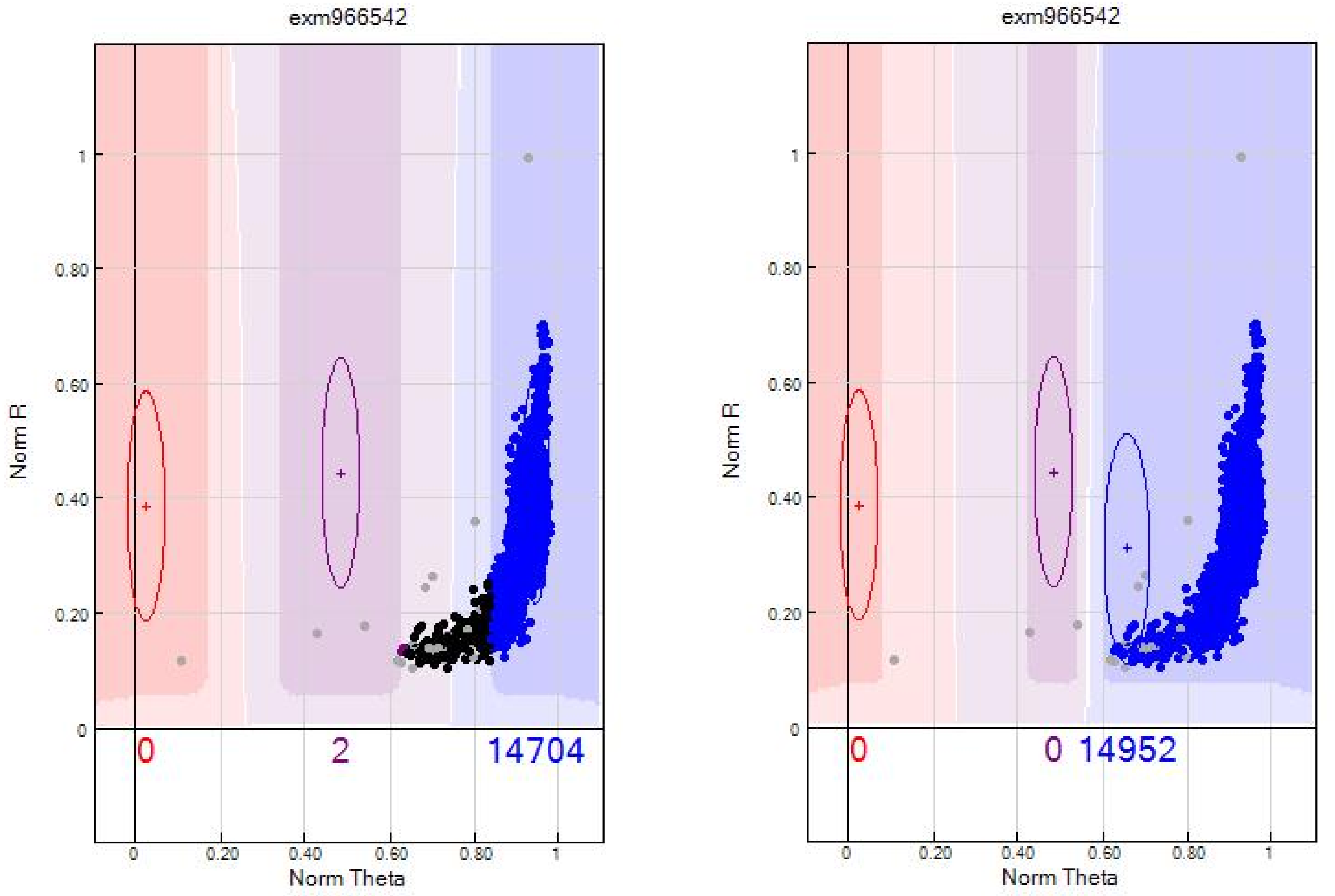
exm966542
Norm R
Norm Theta
0
2
14704
exm966542
Norm R
Norm Theta
0
0
14952

(c)

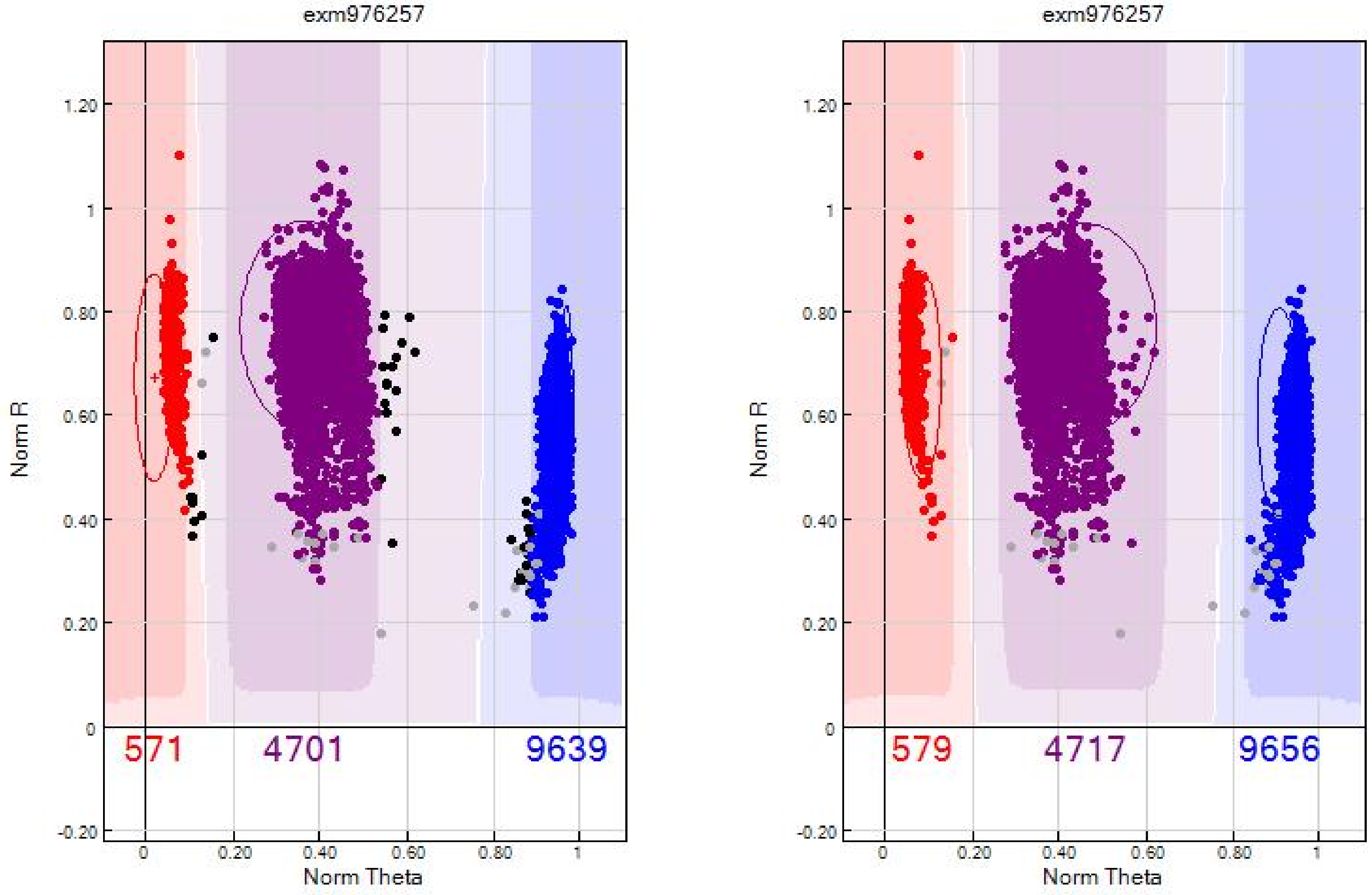
exm976257
Norm R
Norm Theta
571
4701
9639
exm976257
Norm R
Norm Theta
579
4717
9656

Supplementary figure legends

Supplementary Figure 1. Distribution of variants in exonic regions using our guidelines. The *x* and *y* axes indicate the chromosome number and ratio of exonic variants, respectively. The red and blue bars represent the ratio of exonic variants whether using baseline/unfiltered data or our guidelines, respectively.

Supplementary Figure 2. Examples of comparison between before and after manual clustering.

The figures show the three typical clusters seen prior to manual re-clustering that the user controlled directly. Figures on the left show the original cluster patterns, whereas those on the right show the modified cluster patterns. Also, clusters were composed of three different genotypes such as AA (red area on the left), AB (purple area in the middle) and BB (blue area on the right).

Supplementary Table 1. Non-reference concordance and accuracy of hetero allele between before and after of manual re-clustering by minor allele count (MAC)

MAC	Non-reference concordance*		Accuracy of hetero allele*	
	Before	After	Before	After
2	0.9940 ± 0.07	0.9985 ± 0.04	0.8904 ± 0.30	0.9599 ± 0.14
3	0.9918 ± 0.09	0.9989 ± 0.02	0.9159 ± 0.27	0.9523 ± 0.14
4	1.0000 ± 0.00	0.9975 ± 0.04	0.9483 ± 0.21	0.9603 ± 0.11
5	0.9993 ± 0.01	0.9989 ± 0.02	0.9115 ± 0.27	0.9584 ± 0.11

*Non-reference concordance and accuracy of hetero allele were designated as mean ± standard deviation (SD).

Supplementary Table 2. The number of variants in each functional category before and after guideline implementation

Functional category	Exome chip contents	Before our guideline	After our guideline
exonic	219,765	62,655	61,813
intergenic	13,197	7,952	7,923
intronic	5,617	5,332	5,314
splicing	1,964	314	304
ncRNA_intronic	782	744	738
3’ UTR	556	453	450
5’ UTR	237	155	154
downstream	184	174	172
ncRNA_exonic	176	118	119
upstream	172	157	157
exonic;splicing	72	19	19
upstream;downstream	22	22	22
ncRNA_UTR3	16	14	14
ncRNA_UTR5	5	4	4
ncRNA_splicing	1	1	1
Total	242,766	78,114	77,204

UTR, untranslated region; ncRNA, non-coding RNA.
